# Glyburide Reduces Bacterial Dissemination in a Mouse Model of Melioidosis

**DOI:** 10.1371/journal.pntd.0002500

**Published:** 2013-10-17

**Authors:** Gavin C. K. W. Koh, Tassili A. Weehuizen, Katrin Breitbach, Kathrin Krause, Hanna K. de Jong, Liesbeth M. Kager, Arjan J. Hoogendijk, Antje Bast, Sharon J. Peacock, Tom van der Poll, Ivo Steinmetz, W. Joost Wiersinga

**Affiliations:** 1 Center for Experimental and Molecular Medicine, Division of Infectious Diseases, Academic Medical Center, University of Amsterdam, Amsterdam, The Netherlands; 2 Warwick Medical School, University of Warwick, Coventry, United Kingdom; 3 Department of Infection and Tropical Medicine, Heartlands Hospital, Birmingham, United Kingdom; 4 Department of Medicine, University of Cambridge, Cambridge, United Kingdom; 5 Mahidol-Oxford Tropical Medicine Research Unit, Mahidol University, Bangkok, Thailand; 6 Friedrich Loeffler Institute of Medical Microbiology, University Medicine Greifswald, Greifswald, Germany; Swiss Tropical and Public Health Institute, Switzerland

## Abstract

**Background:**

*Burkholderia pseudomallei* infection (melioidosis) is an important cause of community-acquired Gram-negative sepsis in Northeast Thailand, where it is associated with a ∼40% mortality rate despite antimicrobial chemotherapy. We showed in a previous cohort study that patients taking glyburide ( = glibenclamide) prior to admission have lower mortality and attenuated inflammatory responses compared to patients not taking glyburide. We sought to define the mechanism underlying this observation in a murine model of melioidosis.

**Methods:**

Mice (C57BL/6) with streptozocin-induced diabetes were inoculated with ∼6×10^2^ cfu *B. pseudomallei* intranasally, then treated with therapeutic ceftazidime (600 mg/kg intraperitoneally twice daily starting 24 h after inoculation) in order to mimic the clinical scenario. Glyburide (50 mg/kg) or vehicle was started 7 d before inoculation and continued until sacrifice. The minimum inhibitory concentration of glyburide for *B. pseudomallei* was determined by broth microdilution. We also examined the effect of glyburide on interleukin (IL) 1β by bone-marrow-derived macrophages (BMDM).

**Results:**

Diabetic mice had increased susceptibility to melioidosis, with increased bacterial dissemination but no effect was seen of diabetes on inflammation compared to non-diabetic controls. Glyburide treatment did not affect glucose levels but was associated with reduced pulmonary cellular influx, reduced bacterial dissemination to both liver and spleen and reduced IL1β production when compared to untreated controls. Other cytokines were not different in glyburide-treated animals. There was no direct effect of glyburide on *B. pseudomallei* growth *in vitro* or *in vivo*. Glyburide directly reduced the secretion of IL1β by BMDMs in a dose-dependent fashion.

**Conclusions:**

Diabetes increases the susceptibility to melioidosis. We further show, for the first time in any model of sepsis, that glyburide acts as an anti-inflammatory agent by reducing IL1β secretion accompanied by diminished cellular influx and reduced bacterial dissemination to distant organs. We found no evidence for a direct effect of glyburide on the bacterium.

## Author Summary

*Burkholderia pseudomallei* infection (also called melioidosis) is a common cause of bacterial infection in Northeast Thailand, where the mortality rate is 43% despite appropriate antibiotic treatment. We showed previously that patients taking glyburide ( = glibenclamide) prior to admission have lower mortality rates and lower levels of inflammation in the blood. In this study, we used a mouse model to better understand the mechanism underlying this observation. In this study, we used a mouse model of diabetes and infected the mice with *B. pseudomallei*. Half the mice were treated with glyburide and half were not. We also performed *in vitro* experiments to find the minimum concentration of glyburide that would inhibit the growth of *B. pseudomallei*. We found that glyburide treatment was associated with reduced inflammation (as measured by the flow of cells into the lungs and by interleukin-1β production) and reduced spread of the bacterium to liver and spleen when compared to untreated controls. There was no direct effect of glyburide on *B. pseudomallei* growth *in vitro* or *in vivo*. Because the effect of glyburide is on the host and not on the bacterium, it is possible that this effect will be seen in other causes of sepsis, not just melioidosis.

## Introduction

Melioidosis is a severe community-acquired sepsis in Southeast Asia, caused by infection with *Burkholderia pseudomallei*, an environmental soil saprophyte [1]. Its northern-most extent is southern China and its southern-most extent is northern Australia. *B. pseudomallei* has a myriad of clinical presentations ranging from acute pneumonia to chronic skin abscess [1]. More than half of patients are bacteremic, around half have pneumonia and mortality approaches 40% [2]. *B. pseudomallei* has recently been classed a tier 1 bioterror threat agent [3] and is a good clinical model of Gram-negative sepsis [4].

Diabetes is the most commonly identified risk factor for sepsis in general [5] and melioidosis in particular [2], where it occurs in around a third of all melioidosis patients. This is consistent with the fact that diabetes is associated with an impaired host response to *B. pseudomallei*: specifically, it impairs cytokine responses [6]–[8] and macrophage killing of intracellular *B. pseudomallei* [9].

Paradoxically, melioidosis patients with a pre-admission diagnosis of diabetes have a lower risk of mortality than patients without diabetes, which appears to contradict our current knowledge of the effect of diabetes on the host. We have shown previously, in a cohort study of 1,160 patients, that this reduction in risk is associated with glyburide use, and does not appear to be a characteristic of diabetes itself [2].

Glyburide USAN ( = glibenclamide rINN) is a K_ATP_-channel blocker and broad-spectrum ATP-binding cassette (ABC) transporter inhibitor used to treat type 2 diabetes. It costs less than USD 0.10 per dose and appears on the World Health Organization (WHO) model list of essential medicines [10]. Glyburide is readily available and is also one of the most frequently prescribed oral drugs for type 2 diabetes worldwide.

The relevant pharmacological mechanism of glyburide in diabetes is the inhibition of K_ATP_-channels in pancreatic β-cells, which leads to the stimulation of insulin secretion. There is, however, evidence from experimental models that glyburide also has a wide range of anti-inflammatory effects [11]. We confirmed the anti-inflammatory effect of glyburide in a whole blood gene expression study of melioidosis patients on glyburide, however, the anti-inflammatory effect we saw was general, without any one pathway predominating [2].

In this study, we used an experimental model of melioidosis to provide insights into mechanisms underlying the effect of glyburide on inflammation, the best described of which is an inhibitory effect on the host inflammasome, an intracellular protein complex present in macrophages that activates caspase 1 and cleaves pro-IL1β and pro-IL18 to their active forms when presented with an appropriate inflammatory stimulus [12]. We show for the first time, in an experimental model of sepsis, that glyburide acts as an anti-inflammatory agent by reducing IL1β secretion, cellular infiltration into the lungs and bacterial dissemination to distant organs.

## Methods

### Ethics

Mouse experiments were performed at the Academic Medical Center (AMC), University of Amsterdam and were approved by the animal experimentation committee of the AMC (number DIX101725). Mouse experiments were performed in accordance with Dutch law (Wet op de dierproven 1996) and European Council directive (86/609/EEC). Bone marrow-derived macrophage experiments were performed at the Friedrich Loeffler Institut für Medizinische Mikrobiologie, Universitätsmedizin Greifswald, in compliance with the relevant laws.

### Murine models of diabetes and melioidosis

The C57Bl/6J substrain (Jackson Laboratories) is a model for diet-induced type 2 diabetes, because it carries a spontaneous 17.8 kb deletion in the nicotinamide nucleotide transhydrogenase gene, *Nnt*, that appeared in the Jackson Laboratories colony some time between 1951 and 1970 [13], [14]. We therefore elected to use the C57Bl/6N (Charles River) substrain for all mouse experiments because that substrain has an intact *Nnt* gene [15]. There is no consensus in the literature over which mouse strain best models the pathology seen in human disease, and both BALB/c and C57Bl/6 mice have been used [16]–[19]. It has been described by multiple authors that killing of intracellular *B. pseudomallei* by BALB/c mice is deficient compared to C57Bl/6 mice [16], [20]–[23], and that this failure to clear the bacterium results in continued stimulation of type 1 cytokine secretion [21]. The reason for this is not known, but Watanabe *et al.* have reported that BALB/c macrophages express lower levels of the lysosomal enzyme, β-glucuronidase, in response to macrophage-activating lipopeptide-2 (a synthetic TLR2 ligand) and to *E. coli* LPS when compared to C57Bl/6 macrophages [24]. In humans, β-glucuronidase deficiency manifests as Sly syndrome, or mucopolysaccharidosis type VII. The potential association of the BALB/c mouse with an inherited human disease should prompt caution in the interpretation of experiments conducted using this strain.

We used a streptozocin model of diabetes, because that model permits a diabetic phenotype to be induced on any background, at any time, and the cohort become diabetic synchronously [25], [26]. Streptozocin 6 mg/ml (Sigma-Aldrich, Zwijndrecht, The Netherlands) was prepared fresh every day in citrate buffer, then passed through a 0.2 µm polyethersulphone filter (VWR 514-0073) and administered intraperitoneally within 60 minutes of preparation. Citrate buffer 50 mmol/l was made by adding 2.76 g sodium citrate (Merck) and 3.28 g citric acid monohydrate (Merck) to 500 ml de-ionised water, then adjusting the pH to 4. Streptozocin solutions are not stable for longer than 12 h and were therefore made up freshly each day [27]. Specified pathogen-free 10-week-old C57Bl/6NCrl mice (Charles River) were made diabetic by injection with streptozocin 60 mg/kg daily for five days [26]; controls were injected with vehicle. Plasma glucose was checked weekly (Bayer Contour meter) by cheek puncture [28]. For 10-week-old mice not treated with streptozocin, glucose was 8.75±1.52 mM (mean±standard deviation, N = 48) and for 16-week-old mice, 9.30±1.64 mM. We defined diabetes as a plasma glucose ≥16.7 mM (N = 48) [26].

*B. pseudomallei* 1026b is a clinical isolate collected in 1993 from a 29-year-old Thai female rice farmer with bacteremia, soft tissue, cutaneous lesions, septic arthritis and splenic abscesses [29]. Mice were infected with ∼6×10^2^ *B. pseudomallei* 1026b intranasally 4–5 weeks after streptozocin treatment (the LD50 for this model is ∼1×10^2^ cfu [30]). This model of melioidosis has been described in detail elsewhere [19], [31]. In order to mimic our previous clinical cohort study, all animals were treated with full-dose antibiotics. All animals received ceftazidime 600 mg/kg (GlaxoSmithKline, Brentford, England) intraperitoneally starting 24 h after inoculation and continued twice daily until sacrifice [32].

Glyburide 50 mg/kg (Sigma-Aldrich, Zwijndrecht, The Netherlands) or vehicle was administered intraperitoneally once daily starting 7 days before inoculation and continued until sacrifice. Glyburide solutions were prepared in 20% dimethylsulfoxide (DMSO); control mice were given 20% DMSO. This dose of glyburide was chosen because it is equivalent to the highest human dose (20 mg daily to a 50 kg Thai male) after taking into account differences in pharmacokinetics (human t_½_≈8 hours, mouse t_½_≈1 hour) [12].

Animals were allowed *ad libitum* access to food (801733 CRM, Tecnilab-BMI) and water, and were group-housed in polycarbonate cages with 12-hour light/dark cycles, temperature 18–22°C, humidity 40–65%. Eight animals from each group were sacrificed 48, 72 and 96 hours after inoculation. At sacrifice, mice were sedated with ketamine/medetomidine intraperitoneally (12.5 mg/20 µg per 100 g weight), then exsanguinated by cardiac puncture. Death was confirmed by cutting the diaphragm. Glucose measurements were obtained prior to sedation as medetomidine itself elevates glucose concentrations. The left lung was lavaged with three 300 µl aliquots of sterile 0.9% saline then collected for culture. Left lung, liver and spleen were homogenized in sterile saline in a ratio of 1 g organ to 4 ml of saline. Organ homogenates, blood and bronchoalveolar lavage fluid (BALF) were plated onto blood agar in serial dilutions to quantify bacterial burdens. The remaining lung homogenate was incubated for 30 minutes with Greenberger lysis buffer then centrifuged at 650× *g* for 10 minutes. The supernatant was passed through a 0.2 µm filter (PVDF, Millex-GV, Millipore) to remove viable bacteria, then stored at −20°C for cytokine measurements. Heparinized blood was centrifuged at 700× *g* for 10 minutes; the plasma was filtered and stored at −20°C pending analysis.

### Assays

Creatinine was measured with a commercial available kit (Sigma-Aldrich, St. Louis, MO) using a Hitachi analyzer (Boehringer Mannheim, Mannheim, Germany) according to the manufacturers' instructions. In lung homogenates and BALF, interleukin (IL) 1β, IL6, IL10, CXCL5 (LIX) and tumor necrosis factor-α (TNFα) were measured by sandwich ELISA (R&D Systems, Oxford, England). Cytokine levels in plasma were measured by particle immunoassay (mouse inflammation cytometric bead array, BD Biosciences, San Jose, California) because of limited sample quantity. Protein concentrations were measured using the DC™ protein assay (Bio-Rad, Hercules, California).

### Glyburide minimum inhibitory concentration

The minimum inhibitory concentration of glyburide was established by broth microdilution and the protocol was adapted from the CLSI guidelines for microdilution assays of non-fastidious organisms [33]. The positive control was sulfamethoxazole 512 µg/ml and the negative control was DMSO 1%. Glyburide was assayed in doubling dilutions from 0.1 to 1000 µM. The culture medium was cation-adjusted Mueller-Hinton broth (Sigma-Aldrich). Antimicrobial solution 50 µl and inoculum 50 µl (*B. pseudomallei* final concentration 5×10^5^ cfu/ml) were pipetted into each well of a flat-bottomed 96-well plate. The final concentration of DMSO in every well was 1%. Cultures were incubated at 37°C (5% carbon dioxide) for 18 hours. Growth was defined as a pellet ≥2 mm or turbidity on visual inspection. The MIC of glyburide was read as the lowest concentration of glyburide that inhibited the growth of *B. pseudomallei*.

### Bone marrow-derived macrophages

Bone marrow-derived macrophages were generated as previously described [23]. In brief, specified pathogen-free C57Bl/6 mice were anesthetized with sevoflurane then sacrificed by cervical dislocation. Tibias and femurs were removed under aseptic conditions; bone marrow was flushed out with Dulbecco's phosphate-buffered saline (PBS) (Invitrogen, Carlsbad, California) then centrifuged at 150× *g* for 10 minutes. Cells were resuspended in RPMI 1640 (Invitrogen) containing Panexin BMM 5% v/v (PAN Biotech, Aidenbach, Germany), 2 ng/ml recombinant murine GM-CSF (PAN Biotech) and 50 µM 2-mercaptoethanol. Cells were cultured for 10 days at 37°C and 5% carbon dioxide. Cultures were washed with PBS to remove non-adherent cells. Adherent cells were then detached with Trypsin-EDTA (PAA Laboratories, Pasching, Austria) and by scraping. Viable cells were counted in a Neubauer hemocytometer by Trypan blue exclusion. Six-well plates were seeded with 650,000 cells per well, then incubated overnight to allow the cells to adhere.

Cells were pre-treated with varying concentrations of glyburide for 60 min prior to infection (final DMSO concentration 0.1%). Controls were treated with vehicle (RPMI 1640+Panexin 5%+DMSO 0.1%). The inoculum was prepared by culturing *B. pseudomallei* 1026b overnight on Columbia agar with 5% sheep blood. Colonies were suspended in PBS to OD595 0.25 (∼1.2×10^8^ cfu/ml). This suspension was then diluted with RPMI 1640 to the required concentration. Inoculum size was verified by plating serial dilutions onto Columbia agar. Cells were infected with bacteria for 30 minutes, then washed twice in PBS to remove extracellular bacteria. RPMI 1640+Panexin 5%+kanamycin 120 µg/ml (∼4× MIC for this strain to kill extracellular bacteria) was added to each well. Cell culture supernatants were harvested 24 hours after infection, then stored at −20°C pending assay. Supernatants were treated with ultraviolet light for 10 minutes prior to assay in order to sterilize them.

### Statistical analyses

Statistical analyses were performed on Stata 11 (StataCorp, College Station, Texas). Bacterial loads and cytokine concentrations were log-normal. Linear regression was used in preference to ANOVA because it reports not just a *p*-value for each group, but also estimates size of difference and permits interaction testing. Models were fit by the method of variance-weighted least-squares, because (unlike ANOVA) the method does not assume homoscedasticity (i.e., it was not assumed that variance is the same at each time point). Responses were not assumed to vary linearly with time. Separate *p*-values were reported for each time point only when justified by a test for interaction. Results from bone marrow-derived macrophage experiments were analysed by repeated measures ANOVA. Graphs were plot in GraphPad Prism 5.0c for Mac OS X (GraphPad Software, San Diego, California).

### Identifiers

IL1β (Swiss-Prot identifier P01584 human and P10749 murine), IL18 (Swiss-Prot ID Q14116 human and P70380 murine); gamma interferon (Swiss-Prot ID P01579 human and P01580 murine); caspase 1 (Swiss-Prot ID P29466 human and P29452 murine); PYCARD (Swiss-Prot ID Q9ULZ3 human and Q9EPB4 murine); NLRP3 (Swiss-Prot ID Q96P20 human and Q8R4B8 murine); Streptozocin (PubChem CID 29327), Glyburide/glibenclamide (PubChem CID 3488). Swiss-Prot online database may be found at www.uniprot.org.

## Results

### Diabetes results in delayed bacterial clearance

**Figure 1:**
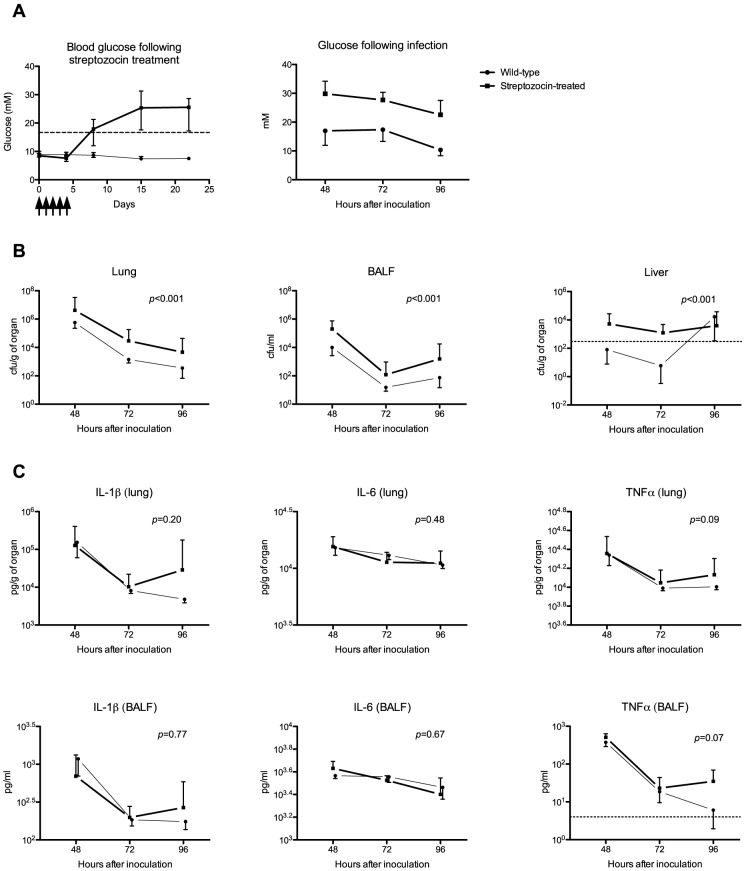
Bacterial loads are higher in diabetes. Note. BALF = bronchoalveolar lavage fluid; cfu = colony-forming units; IL = interleukin; STZ = streptozocin; TNFα = tumor necrosis factor-α. Sixty mice were injected with STZ 60 mg/kg daily for five days (marked by five vertical arrows on horizontal axis) and compared to 60 controls (treated with citrate buffer pH 4). Only 48 animals were eventually used for infection experiments. A. Glucose levels in streptozocin-treated animals plateaued 14 days after the first dose of streptozocin was given. The error bars in the graph are ranges. The horizontal interrupted line is the 16.7 mM cut off used to define diabetes in mice: streptozocin-treated animals treated were not used if their blood glucose concentrations fell below this level. The graph on the right shows glucose concentrations at time of sacrifice, and the error bars shown as standard deviations. B. Animals were inoculated with ∼6×10^2^ cfu *B. pseudomallei* 4–5 weeks after streptozocin treatment. All mice were treated with ceftazidime starting 24 h after inoculation and continued until sacrifice at 48, 72 or 96 h (8 animals per group per time point). Bacterial loads were higher in lung, BALF and liver in diabetic animals. Error bars are 95% confidence intervals of the mean. C. When comparing diabetic animals with controls, no differences were seen in any of the cytokines measured.

In our murine model of diabetes blood glucose levels began to rise one week after the fifth streptozocin dose was given and reached a plateau after a further week (Figure 1A left). Losses in the streptozocin-treated group were ∼20% with this regimen, either from complications of the hyperglycemia or because the animals failed to become diabetic and had to be sacrificed. The mean blood glucose concentration at sacrifice was 26.7 mM in diabetic mice compared to 14.9 mM in controls (Figure 1A right). Mean plasma creatinine concentration was 10.4 µM in streptozocin-treated mice, compared to 6.1 µM in untreated controls (*p* = 0.05, Welch t-test).

Having established a murine model of diabetes, we proceeded to examine the influence of diabetes on the pathogenesis of melioidosis. We infected diabetic mice with ∼6×10^2^ *B. pseudomallei* intranasally. So as to mimic the clinical situation more closely, all animals received ceftazidime treatment starting 24 h after inoculation (which is the point at which the animals first become symptomatic [19]).

Mice were sacrificed after 48, 72 and 96 h (i.e., when animals showed signs of recovery following ceftazidime treatment) to determine bacterial loads in lungs and BALF (the primary site of the infection), liver, spleen and blood (to evaluate dissemination to distant body sites; Figure 1). We found that bacterial loads were higher in lung, BALF and liver of diabetic mice when compared to non-diabetic controls (Figure 1B). There were non-significant trends to higher bacterial loads in diabetic mice compared to non-diabetic controls in spleen and blood (data not shown). Having found that diabetes delays bacterial clearance, we sought to characterize the host response to infection by measuring cytokine responses in lung and BALF. We found no differences in cytokine responses in lung or BALF (IL1β, IL6, TNFα, Figure 1C) despite the higher bacterial loads in diabetic mice compared to non-diabetic controls. Since cytokine responses and bacterial loads are usually correlated, this suggests that a state of relative immunosuppression exists in diabetes.

### Glyburide does not directly inhibit the growth of *B. pseudomallei*

Glyburide is structurally related to the sulfonamides, which are active against *B. pseudomallei*. If glyburide inhibits the growth of *B. pseudomallei*, then the differences in the inflammatory response we observed in our clinical study [2] might be explained by a direct effect on the bacterium and not by an effect on the host response. In order to exclude the possibility that glyburide directly inhibits growth of *B. pseudomallei*, we measured the minimum inhibitory concentration (MIC) of glyburide, which we found to be >1000 µM (more than three orders of magnitude higher than the highest concentration achievable in humans [34]). It therefore seems unlikely that glyburide is acting via a direct effect on the bacterium.

### Glyburide does not reduce glucose concentrations in the streptozocin model of diabetes

We proceeded to examine the effect of glyburide on the host response to melioidosis, using the model of diabetes just described.

The host response is altered by changes in glucose concentration. The primary action of glyburide in diabetes is to reduce blood glucose concentration by stimulating insulin secretion by the β-islet cells of the pancreas. Streptozocin treatment destroys the β-cells of the pancreas, making animals hyperglycemic and unresponsive to the action of glyburide. This allowed us to dissect out the separate effects of glyburide on glucose and on the inflammatory response. Blood glucose measurements taken at sacrifice confirmed that in our model of diabetes, blood glucose concentrations are indeed unaltered by glyburide treatment compared to vehicle-treated controls (mean glucose 23.9 mM in glyburide-treated animals versus 21.8 mM in untreated controls, *p* = 0.40).

### Glyburide reduces systemic dissemination of *B. pseudomallei*

**Figure 2:**
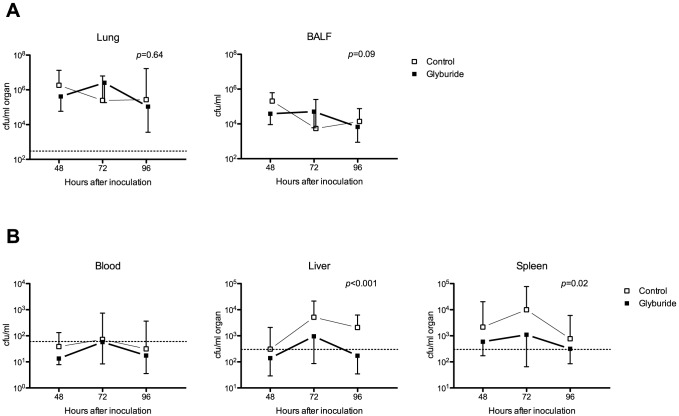
Bacterial loads in blood, liver and spleen are lower in glyburide-treated mice. Note. BALF = bronchoalveolar lavage fluid; cfu = colony-forming units. Mice were treated with glyburide or vehicle for seven days prior to inoculation with ∼6×10^2^ cfu *B. pseudomallei*. All mice were treated with ceftazidime starting 24 h after inoculation and continued until sacrifice at 48, 72 or 96 h (eight animals per group per time point). Error bars are standard deviations. The horizontal interrupted line marks the limit of detection. **A.** There were no differences found in bacterial loads at the primary site of infection (lungs and BALF). **B.** Bacterial loads in glyburide-treated animals were lower in liver and spleen compared to untreated controls. In blood, there was a trend to bacterial loads being lower in glyburide-treated animals compared to untreated controls, but no *p*-value is reported because a large number of points were below the limit of detection (3 cfu/50 µl).

In this model of melioidosis pneumonia, the primary site of infection is the lungs. We found no effect of glyburide on bacterial loads in either lung tissue or in BALF at any of the three time points measured (*p* = 0.64 and *p* = 0.09 respectively; Figure 2A). However, glyburide reduced the systemic dissemination of *B. pseudomallei* from the lungs to the systemic compartment: bacterial loads were lower in liver and spleen all three time points measured in the glyburide treated animals when compared to control (Figure 2B).

### Glyburide is associated with reduced cellular influx into the lungs

To examine the mechanisms underlying the reduced bacterial dissemination following glyburide treatment, we next investigated the influence of glyburide on the pulmonary inflammatory response during experimental melioidosis.

**Figure 3:**
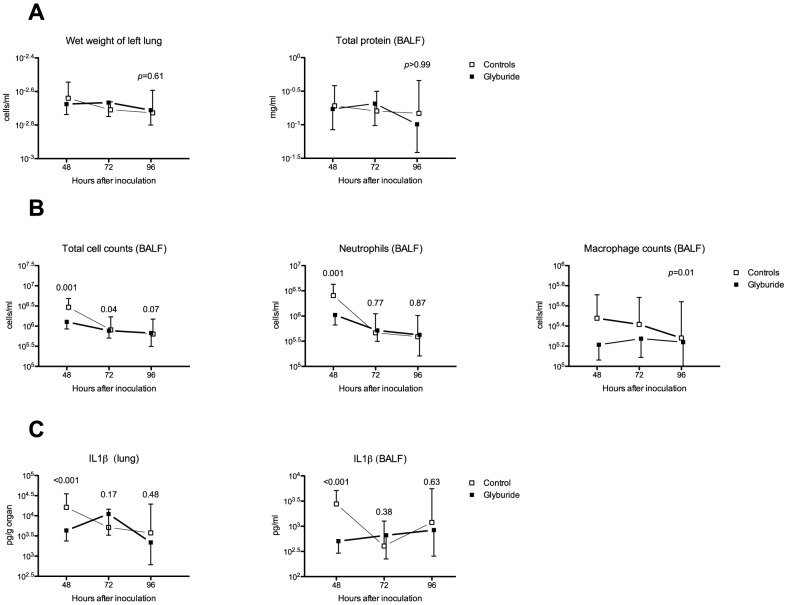
Diminished pulmonary leukocyte influx and IL1β production in glyburide-treated mice. Note. BALF = bronchoalveolar lavage fluid; IL1β = interleukin-1beta. Mice were treated with glyburide or vehicle for seven days prior to inoculation with ∼6×10^2^ cfu *B. pseudomallei*. All mice were treated with ceftazidime starting 24 h after inoculation until sacrifice (eight animals per group per time point). Error bars indicate standard deviations. A single *p*-value is reported unless there is evidence from a test of interaction that effects at each time point are different. **A.** No differences were found in wet weight of lung or in protein infiltration. **B.** Cellular infiltrate was reduced in glyburide-treated mice compared to untreated controls at the 48-hour time point and this difference was seen in both neutrophil and monocyte fractions. **C.** IL1β concentrations were lower in glyburide-treated mice compared to untreated controls at the 48-hour time point. This difference was greater in BALF than in lung tissue.

Melioidosis pneumonia is characterized by a florid influx of cells into the lungs, and these cells are predominantly neutrophils [19]. Although neutrophils form a critical part of the innate host response to melioidosis [35], neutrophils may also contribute to mortality by causing damage to the respiratory tract and have been implicated in the pathogenesis of the acute respiratory distress syndrome [36]. In this study, we found that glyburide did not influence the wet weight of the inflamed lungs or the amount of protein in BALF (both are markers for capillary leakage; Figure 3A). We did, however, find that the total number of cells was reduced in glyburide-treated animals compared to controls. The majority of cells in this inflammatory infiltrate were neutrophils, but macrophages were also present in significant numbers. This difference in cell counts was most apparent at the 48 h time point (Figure 3B).

### Glyburide is associated with lower IL1β concentrations in lung

Glyburide has previously been reported to inhibit inflammasome assembly and the key function of the NLRP3 inflammasome is the activation and secretion of IL1β [12]. Since IL1β has been implicated in the recruitment of neutrophils into the inflamed lung, we hypothesized that glyburide might limit the influx of cells via a reduction in IL1β release [37], [38]. In line with this hypothesis, we found that in glyburide-treated animals, concentrations of IL1β were 73.3% lower in lung tissue 48 hours after inoculation compared to controls (*p*<0.001, Figure 3C). In lung tissue, IL1β is present in both its mature and immature forms, and only mature IL1β is secreted into the alveolar space. IL1β in bronchoalveolar lavage fluid (BALF) should, therefore, be a better measure of inflammasome activation than lung tissue. We found that IL1β concentrations in BALF were 81.7% lower in glyburide-treated mice than controls (*p*<0.001, Figure 3C) at 48 hours.

**Figure 4:**
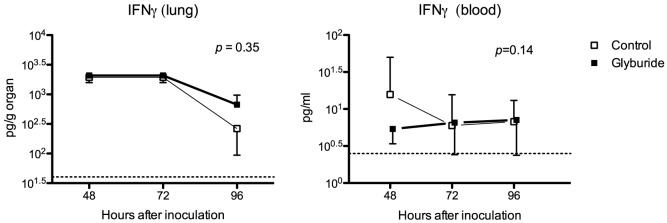
Glyburide does not influence production of IFNγ. Note. IFNγ = gamma interferon. Mice were treated with glyburide or vehicle for seven days prior to intranasal inoculation with ∼6×10^2^ *B. pseudomallei*. All mice were treated with ceftazidime starting 24 h after inoculation and continued until sacrifice (eight animals per group per time point). Error bars indicate standard deviations. A horizontal interrupted line marks the limit of detection for the assay. No influence of glyburide on IFNγ responses in both the pulmonary and systemic compartment was found.

Another role of the NLRP3 inflammasome is the activation and secretion of IL18, which stimulates natural killer and CD8^+^ cells to secrete gamma interferon (IFNγ). Inflammasome inhibition would be expected to inhibit the secretion of mature IL18, and in turn reduce IFNγ secretion [39]. Immature IL18 is constitutively expressed at high concentrations in unstimulated cells [40]. We therefore did not measure IL18 in lung tissue. Instead, IL18 was measured in BALF, which should represent the mature secreted form of the cytokine only. At the 48-hour time point, we found no difference in IL18 concentrations (geometric mean 251 pg/ml in the glyburide-treated animals versus 401 pg/ml in control animals, *p* = 0.17, results not shown). We also found no differences in IFNγ concentrations in lung or in blood (Figure 4).

### Glyburide has no effect on cytokines unrelated to the inflammasome

In order to determine if the effect of glyburide was isolated to IL1β, or whether it was a general anti-inflammatory effect, we assayed the pro-inflammatory cytokines, IL6, TNFα and CXCL5. The secretion of these three cytokines is unrelated to the inflammasome. We also considered the possibility that glyburide might be acting to promote secretion of the anti-inflammatory cytokine, IL10. Although there were differences in IL6 and TNFα at the 48 h time point, these differences were small and did not reach statistical significance. Overall, we found no evidence of an effect of glyburide on the secretion of CXCL5, IL6, IL10 or TNFα (Figure S1).

### Glyburide reduces IL1β production in bone marrow derived macrophages

**Figure 5:**
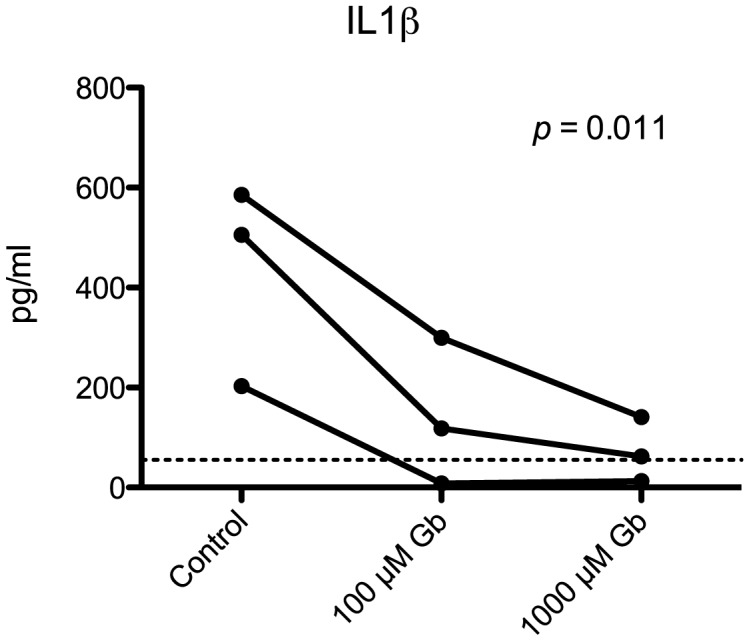
Glyburide reduces macrophage IL1β secretion. Note. Gb = glyburide; IL = interleukin. Primary bone marrow-derived macrophages were infected with *B. pseudomallei* for 30 min (multiplicity of infection 25 cfu/cell) and incubated with 0 µM (control), 100 µM or 1000 µM glyburide. IL1β was assayed in supernatant 24 hours later. The graph shows the results of three separate experiments, each linked by solid lines. The limit of detection for this assay was 55.2 pg/ml.

To confirm that that glyburide was acting directly on macrophage secretion of IL1β, we infected primary bone marrow-derived macrophages with *B. pseudomallei* and treated the cells with varying concentrations of glyburide. In correspondence with our in vivo data, we found that glyburide reduced IL1β secretion in a dose-dependent fashion (Figure 5).

## Discussion

We have developed a murine model of diabetes and shown that it is more susceptible to melioidosis than wild-type animals, as evidenced by higher bacterial loads. This adds to the growing body of evidence that diabetes *per se* increases susceptibility to melioidosis and direct effects of diabetes are not responsible for the improved survival seen in our previously reported clinical study [2]. Hodgson *et al.* report increased mortality from subcutaneous *B. pseudomallei* infection in a murine model of diabetes (BKS.Cg-Dock7(m)+/+Lepr(db)/J mice), and found a defect in intracellular killing by peritoneal macrophages taken from diabetic animals compared to non-diabetic animals [9]. The reason behind the increased susceptibility in diabetes is unclear, but may relate to delayed or reduced cytokine production in diabetes [7], [8], [41].

We next presented evidence for an anti-inflammatory effect of glyburide in a diabetic mouse model of melioidosis and this anti-inflammatory effect appears specific to IL1β. This was supported by data from stimulated BMDMs, in which glyburide reduced IL1β secretion in a dose dependent fashion. This is consistent with the results of previous *in vitro* and *in vivo* reports [12], [42], [43]. To the best of our knowledge, this is the first time an inhibitory effect of glyburide on IL1β secretion has been described in any model of sepsis.

We hypothesized that the effect of glyburide was independent of its effect on glucose control. This is because in our previously reported clinical study [2], both patients with hyperglycemia and patients who were never hyperglycemic had higher mortality rates than diabetic patients treated with glyburide, suggesting that the effect we were seeing was unrelated to hyperglycemia, even though alterations to plasma glucose and insulin concentrations are themselves well-known to cause changes to the host response [5]. Although the majority of cases of diabetes in Thailand have type 2 diabetes, we therefore elected to use a model of diabetes in which glucose concentrations were not responsive to glyburide, so as to answer the question raised by our clinical study.

A limitation of clinical studies of infection has always been that the interplay between the burden of infection and the inflammatory response has been difficult to tease out. Cytokine levels may be higher because bacterial burdens are higher, or bacterial burdens may be higher because of an ineffective innate host response [44]. Animal studies allow us to control the size of the initial inoculum and therefore the initial burden of infection. In this study, we found that bacterial loads in the lungs (the primary site of infection in our model) were not different between glyburide-treated and untreated mice. This excludes the possibility that the differences in IL1β levels in the lungs are due to an effect of glyburide on the burden of infection in the lungs.

We found that glyburide limits the cellular influx into the lungs, with effects on both neutrophil and monocyte numbers. Neutrophils are a crucial part of the host response to melioidosis [35], but neutrophils have also been implicated in the pathogenesis of the acute respiratory distress syndrome [36]. Glyburide was only associated with a reduction in the magnitude of the neutrophil response and not its complete ablation (neutrophils are not a normal constituent of BALF taken from the healthy lung). This balance between ‘enough’ and ‘too much’ is a common theme in the pathogenesis of sepsis and its complications [45]. A reduction in neutrophil influx into the lungs is consistent with our finding that glyburide is associated with a lower incidence of respiratory failure in patients with melioidosis [2].

The host response to *B. pseudomallei* is critically dependent on cellular immunity [46]. Macrophages, in particular, play a critical role in initiating the host cytokine response [47]–[49], in inflammasome activation [50] and in recruiting neutrophils to the site of inflammation (by secreting IL1β [37], [51] and chemo-attractants such as IL8). Paradoxically, *B. pseudomallei* is a facultative intracellular pathogen that is capable of parasitizing macrophages [52]–[54]. Bacterial burdens in blood, liver and spleen were lower in our model, suggesting that glyburide reduces bacterial dissemination. The role of neutrophils and macrophages in the dissemination of *B. pseudomallei* is not known, but it is possible to speculate that the reduced influx of leukocytes into the lungs reduces the number of cells parasitized by the bacterium and therefore impairs the ability of *B. pseudomallei* to disseminate [55].

We found no effect on cytokines unrelated to the inflammasome. Specifically, there was no effects seen on CXCL5, IL6, IL10 or TNFα secretion, which is again consistent with previous reports in the literature [11], [12] and rules out a more general anti-inflammatory effect. In this model, all animals were treated with full-dose ceftazidime in order to simulate the clinical study that motivated this experiment [2]. Under this regimen, all animals showed signs of recovery by 96 hours and the fact that no differences were seen in cytokine responses at later time points is therefore not surprising.

The mechanism by which glyburide is able to inhibit IL1β secretion is not known. Lamkanfi *et al.* demonstrated that glyburide is able to inhibit assembly of the NLRP3 inflammasome in response to stimulation with lipopolysaccharide (LPS) and adenosine triphosphate (ATP) [12]. However, when *Salmonella enterica* serovar Typhimurium was used as a stimulus, glyburide was not able to prevent inflammasome assembly, as measured by caspase 1 cleavage (caspase 1 being a critical component of the NLRP3 inflammasome) [12]. This suggests that there are alternative pathways which permit the assembly of the NLRP3 inflammasome. The pathway that glyburide blocks when macrophages are confronted by a complex stimulus such as a whole bacterium is not known.

Our IL1β assay does not distinguish between immature pro-IL1β and mature cleaved IL1β. The concentrations of IL1β we report in lung tissue therefore represent a combination of newly synthesised immature IL1β and mature IL1β. However, only mature IL1β is secreted into BALF and it therefore a marker of inflammasome assembly. In our model, glyburide-treatment reduced IL1β secretion in BALF, but did not block it completely. This means that there must have been some inflammasome assembly despite glyburide treatment. Immature IL18 is also cleaved by the inflammasome, so our finding that inflammasome assembly is not completely blocked by glyburide in melioidosis is supported by the fact that we observed a slight but non-significant reduction in IL18.

We know that inflammasome-activation is necessary for the host response to *B. pseudomallei* infection [50], so it seems implausible that inflammasome-inhibition would be protective in clinical melioidosis. Although we and others have previously reported that IL18 protects against *B. pseudomallei* lung infection [56], Ceballos-Olvera *et al.* recently demonstrated that the IL1β response appeared to be harmful to the host [57]. Our finding that glyburide seems to preferentially inhibit a potentially deleterious IL1β response while preserving the IL18 response would therefore be consistent with a beneficial effect of glyburide in clinical disease.

Our study does not exclude other mechanisms for the action of glyburide. Aside from an effect on the inflammasome, Hamon *et al.* have presented evidence that glyburide may block the secretion of mature IL1β by an inflammasome-independent mechanism [11]. Glyburide has also been shown to have other anti-inflammatory effects, including the prevention of ischemic reperfusion injury [58], [59], and an enhancement of intracellular killing of *Leishmania* parasites [60].

In conclusion, we have replicated our clinical finding that glyburide treatment exerts beneficial effects in melioidosis [61], using an experimental model of melioidosis in diabetic mice. We now show for the first time in any model of sepsis that glyburide acts as an anti-inflammatory agent by reducing IL1β secretion, cellular infiltration into the lungs and bacterial dissemination to distant organs. Although glyburide has previously been shown to prevent IL1β maturation and secretion by inhibiting inflammasome assembly, the presence of still detectable levels of IL1β in BALF, coupled with smaller-than-expected effects on IL18 and IFNγ, means inflammasome inhibition is incomplete, or that this may only explain partly the mechanism by which glyburide acts in the context of melioidosis.

## Supporting Information

Figure S1 **Glyburide does not influence production of non-inflammasome cytokines in pulmonary** ***B. pseudomallei*** **infection.** Note. BALF = bronchoalveolar lavage fluid; IL = interleukin; TNFα = tumor necrosis factor-alpha. Mice were treated with glyburide or vehicle for 7 days prior to inoculation with ∼6×10^2^ cfu *B. pseudomallei*. All mice were treated with ceftazidime starting 24 h after inoculation until sacrifice (8 animals per group per time point). Error bars indicate standard deviations. A single *p*-value is reported for each cytokine unless there is evidence from a test of interaction that effects at each time point are different. A horizontal interrupted line marks the limit of detection for the assay. Glyburide did not influence the production of IL6, IL10, CXCL5 and TNFα in the systemic or pulmonary compartment.
(EPS)
